# Persistent Long-Term Risk After Primary Surgery for Head and Neck Adenoid Cystic Carcinoma: Competing-Risk and Conditional Estimates

**DOI:** 10.3390/cancers18050833

**Published:** 2026-03-04

**Authors:** Ivica Lukšić, Marko Tarle, Marina Raguž, Petar Suton

**Affiliations:** 1 Department of Maxillofacial and Oral Surgery, Dubrava University Hospital, 10000 Zagreb, Croatia; luksic@kbd.hr (I.L.); mtarle@kbd.hr (M.T.); 2 School of Medicine, University of Zagreb, 10000 Zagreb, Croatia; 3 School of Dental Medicine, University of Zagreb, 10000 Zagreb, Croatia; 4 Department of Neurosurgery, Dubrava University Hospital, 10000 Zagreb, Croatia; maraguz@kbd.hr; 5 School of Medicine, Catholic University of Croatia, 10000 Zagreb, Croatia; 6 Department of Oncology and Radiotherapy, Dubrava University Hospital, 10000 Zagreb, Croatia; 7 Faculty of Medicine, University of Rijeka, 51000 Rijeka, Croatia

**Keywords:** adenoid cystic carcinoma, head and neck cancer, salivary gland neoplasms, long-term follow-up, survival analysis

## Abstract

**Background/Objectives**: Head and neck adenoid cystic carcinoma (HNAdCC) is characterized by indolent growth but sustained long-term risk of late recurrence and disease-related mortality. Data describing very long-term outcomes using analytic approaches that explicitly account for competing mortality remain limited. We aimed to characterize late failures, competing causes of death, and clinically interpretable long-horizon risk estimates after primary surgery for HNAdCC. **Methods:** We performed a retrospective single-center cohort study of patients with HNAdCC treated with curative-intent surgery between 1984 and 2020. Overall survival (OS) and cancer-specific survival (CSS) were estimated using Kaplan–Meier method. Competing risks of disease-related and other-cause death, as well as first-failure patterns, were analyzed using cumulative incidence functions, including a 5-year landmark analysis. Conditional mortality and restricted mean survival time (RMST; τ = 25 years) were additionally assessed. **Results:** Fifty-seven patients were included (median age 54 years). Median follow-up was 133 months overall and 212 months among survivors. A first failure occurred in 19/57 (33.3%) of patients, with distant metastasis as the most common pattern; 7/19 (36.8%) of failures occurred beyond 5 years. OS at 5, 10, and 25 years was 68.4%, 64.9%, and 37.5%, respectively; corresponding CSS was 78.9%, 74.8%, and 51.7%. At 25 years, cumulative incidence of disease-related death was 41.7%, compared with 20.9% for other-cause death. Older age and advanced T category were independently associated with worse OS, while older age and perineural invasion predicted worse CSS. Among 5-year survivors, conditional risk of disease-related death by 25 years remained 32.7%. RMST analyses demonstrated substantial long-term life-years lost associated with perineural invasion and T3–4 disease. **Conclusions:** HNAdCC exhibits persistent long-term risk with clinically meaningful late failures and substantial competing mortality over decades. Conditional and RMST-based estimates provide patient-centered measures that support lifelong, risk-adapted surveillance, particularly focused on detection of distant metastases.

## Simple Summary

Head and neck adenoid cystic carcinoma is uncommon and often grows slowly, but it can return or spread many years after treatment. We reviewed 57 patients treated with curative surgery at our hospital between 1984 and 2020 and followed them for up to 40 years. We found that about one in three patients developed a first recurrence or metastasis, and more than one in three of these events happened later than 5 years after surgery. When the cancer spread, it most often involved the lungs. Over a very long follow-up, deaths caused by cancer remained frequent, while deaths from other (non-cancer) causes also accumulated and needed to be considered when estimating long-term risk. Older age, more advanced local tumor stage, and tumor spread along nerves were associated with worse outcomes. These results show that patients cannot be considered “out of danger” after 5 years and support lifelong, risk-adapted follow-up, with particular attention to detecting lung metastases.

## 1. Introduction

Adenoid cystic carcinoma (AdCC) is a rare malignancy, accounting for approximately 1% of head and neck cancers and 10–15% of malignant salivary gland tumors. While WHO summaries often describe mucoepidermoid carcinoma as the most common malignant salivary gland tumor, some European single-center cohorts report AdCC as the most frequent malignancy, particularly among minor salivary glands (e.g., 30.5% of malignant tumors; 73/239 in a large Polish series) [1,2]. It arises predominantly from major and minor salivary glands but can also originate from other gland-bearing sites, including the sinonasal tract, lacrimal gland, and tracheobronchial tree [2,3,4]. Although AdCC often demonstrates slow initial growth, it exhibits infiltrative behavior and marked neurotropism, with perineural invasion (PNI) reported in 50–70% of cases, as well as a propensity for hematogenous dissemination [2,3,4,5]. This biology results in a distinctive outcome pattern: five-year survival is relatively favorable, yet long-term survival declines steadily over decades, reflecting a persistent “long tail” of late recurrence and disease-related mortality [4,5]. Seminal reports such as Eneroth et al. (1968) highlighted decades-long follow-up with very late recurrences in palate AdCC, and subsequent institutional and population-based studies (e.g., Mendenhall et al., 2004; Ellington et al., 2012) continued to document ongoing long-term survival attrition beyond 10–15 years [6,7,8]. Molecular data further underscore biological heterogeneity: MYB/MYBL1 alterations represent early oncogenic events, while NOTCH pathway alterations enriched in recurrent or metastatic disease (approximately 20–30%) and rare high-grade transformation are associated with more aggressive behavior and poorer outcomes [2,9,10].

For localized head and neck adenoid cystic carcinoma (HNAdCC), complete surgical resection is the cornerstone of curative-intent treatment, while postoperative radiotherapy (PORT) is commonly recommended in the presence of adverse features such as advanced T stage, close or positive surgical margins, PNI, bone invasion, or high-grade histology [11,12,13]. Although PORT improves locoregional control, its effect on overall survival remains inconsistent, and durable cure is often limited by late distant metastasis that can occur after prolonged disease-free intervals exceeding 5–10 years [11,14,15,16]. In long-term cohorts, the lung is the predominant metastatic site, followed by bone and liver [14,15,17]. A large systematic review and meta-analysis including 17,497 patients reported overall survival rates of approximately 74% at five years, 49% at ten years, and 27% at twenty years, underscoring continuous attrition over time rather than a survival plateau [16]. In the recurrent or metastatic setting, systemic treatment options remain limited, with modest response rates and largely palliative benefit; consequently, long-term survivorship care depends on quantifying late, time-dependent risks and appropriately accounting for competing mortality during extended follow-up when clinically relevant endpoints compete [14,15,16].

Survivorship recommendations therefore emphasize prolonged surveillance, yet practical implementation varies considerably between institutions, and there remains limited high-quality evidence defining how long and how intensive follow-up should be for a given risk profile [18]. In this context, analytic approaches that explicitly account for competing risks and deliver time-updated, patient-centered prognostic information may be particularly valuable. Conditional estimates, risk assessments updated for patients who have already remained event-free for a given number of years, can address the common clinical question faced by long-term survivors: “What is my risk from this point forward?” In parallel, interpretable measures such as restricted mean survival time (RMST) can complement hazard-based summaries by quantifying absolute time gained or lost over clinically relevant horizons, an approach increasingly advocated in settings where proportional hazards may be doubtful or where long-term tails of risk are expected [19].

Against this background, we analyzed a single-center cohort of surgically treated HNAdCC with very long follow-up to characterize long-term survival outcomes, describe the timing and pattern of first failure, and quantify competing mortality and post-failure outcomes using methods designed for extended survivorship trajectories. By integrating competing-risk and conditional frameworks and reporting clinically interpretable long-horizon measures, our study aims to provide more actionable risk estimates for counseling, survivorship planning, and the design of future follow-up strategies and clinical studies.

## 2. Materials and Methods

### 2.1. Study Design, Setting, and Reporting

We conducted a retrospective, single-center cohort study at Dubrava University Hospital (Zagreb, Croatia), a tertiary referral center. The study was designed to characterize very long-term outcomes after primary surgery for HNAdCC, with emphasis on overall and cancer-specific survival, patterns and timing of first failure, and competing mortality over decades of follow-up. The study is reported in accordance with STROBE recommendations for observational cohort studies (Supplementary Figure S1).

### 2.2. Patient Identification and Eligibility Criteria

Institutional medical records were screened to identify consecutive patients diagnosed with histologically confirmed HNAdCC between August 1984 and December 2020. During this period, 80 patients were diagnosed with HNAdCC. Patients were eligible for inclusion if they underwent primary curative-intent surgical treatment at our institution and had sufficient clinical and follow-up information to ascertain survival status and major outcome events. Patients were excluded if they received non-surgical primary treatment (e.g., definitive radiotherapy for surgically unresectable and/or medically inoperable disease), underwent primary surgery outside our institution with insufficient operative/pathological documentation, or lacked essential follow-up data required for time-to-event analyses. The final analytic cohort comprised 57 patients.

### 2.3. Data Collection and Variables

Clinical, pathological, treatment, and follow-up data were retrospectively abstracted from hospital charts and entered into a structured database. Variables included: (a) Demographics: age at diagnosis/surgery and gender. (b) Tumor characteristics: primary site and origin (major vs. minor salivary gland), pathological T category and N category when available, pathological stage, largest tumor dimension (when available), and adverse histopathological features including perineural invasion (PNI), perivascular invasion (PVI), surgical margin status, and extranodal extension (ENE) in node-positive cases. All cases were restaged according to American Joint Committee on Cancer (AJCC) Cancer Staging Manual, 8th Edition. Site-specific AJCC criteria were applied. Tumors arising from the palate/maxillary gingiva were staged using the oral cavity classification; in this setting, invasion of the maxillary bone and/or limited extension into the maxillary sinus was categorized as T4a. Tumors with a primary epicenter within the maxillary sinus or other paranasal sinus subsites were staged using the corresponding sinonasal criteria. (c) Treatment variables: type and extent of primary surgery, performance of neck dissection (if applicable), and adjuvant therapy details (radiotherapy). For radiotherapy, dose was recorded when available.

### 2.4. Treatment and Follow-Up Strategy

All included patients underwent primary curative-intent surgery. Decisions regarding neck dissection and adjuvant radiotherapy were made by a multidisciplinary tumor board and evolved over the study period. Postoperative radiotherapy was delivered in selected patients according to risk-adapted institutional practice; no patient received concurrent chemotherapy. Radiation dose (Gy) was recorded when available. Follow-up was performed according to institutional practice with clinical examinations and imaging as clinically indicated and was administratively closed on 1 March 2025.

### 2.5. Outcome Definitions

Follow-up time (months) was calculated from the date of primary surgery to the date of last clinical contact or death. Vital status at last follow-up was classified as: alive without disease, alive with disease, dead from HNAdCC, or dead from other causes. Cause of death was determined from available clinical records and documentation in the medical chart. Overall survival (OS) was defined as the time from primary surgery to death from any cause. Patients alive at last follow-up were censored at the date of last contact. Cancer-specific survival (CSS) was defined as the time from primary surgery to death attributable to HNAdCC. Deaths from other causes were censored at the time of death for Kaplan–Meier CSS analyses. Patterns of disease failure were recorded based on the first documented event after completion of primary treatment and classified as: local recurrence, regional nodal recurrence, distant metastasis, or combined local plus regional recurrence. Time to first failure was calculated from the date of primary surgery to the date of radiologically and/or histologically confirmed recurrence/metastasis. Residual disease detected immediately after completion of primary therapy was documented separately and was not classified as recurrence. Salvage surgery was defined as any surgical intervention performed for recurrent disease, residual disease, or metastatic disease after primary treatment.

### 2.6. Statistical Analysis

Continuous variables are summarized as mean ± SD or median (IQR), and categorical variables as *n* (%). Overall survival (OS) and cancer-specific survival (CSS) were estimated using the Kaplan–Meier method and compared with the log-rank test where applicable. OS was calculated from the date of surgery to death from any cause; CSS from surgery to death attributable to HNAdCC, with other-cause deaths censored for Kaplan–Meier CSS. Prognostic factors for OS and CSS were evaluated using Cox proportional hazards regression; multivariable models were kept parsimonious given the limited number of events, and age was modeled per 10-year increment. The proportional hazards assumption was assessed using standard diagnostics (e.g., Schoenfeld residuals). Given the long follow-up and non-trivial competing mortality, cumulative incidence functions (Aalen–Johansen) were used to estimate absolute risks of disease-related death and other-cause death, and to quantify first-failure patterns in the presence of competing events (death without a documented prior failure). Patients with immediate post-treatment residual disease were analyzed separately and excluded from first-failure cumulative incidence analyses. A prespecified 5-year landmark analysis was performed among patients alive and event-free at 5 years, with time re-zeroed at the landmark. Conditional survival over intervals was calculated as S(t + s)/S(t). Restricted mean survival time (RMST) for OS was estimated up to τ = 25 years, with between-group differences summarized as absolute time gained/lost. Statistical analyses were performed using MedCalc, v12.5.0 (MedCalc Software, Ostend, Belgium; https://www.medcalc.org, accessed on 15 January 2026). Two-sided *p* values < 0.05 were considered statistically significant.

### 2.7. Ethical Approval

The study was approved by the Ethics Committee of Dubrava University Hospital (2025/0904-2, 4 September 2025). The requirement for written informed consent was waived due to the retrospective design and use of routinely collected clinical data. All data were anonymized prior to analysis. The study was conducted in accordance with the Declaration of Helsinki and applicable data protection regulations.

## 3. Results

### 3.1. Patient and Tumor Characteristics

A total of 57 patients with HNAdCC treated with primary surgery were included. Median age at diagnosis was 54 years (IQR 43–63), and 30/57 (52.6%) were female. Tumors originated from minor salivary glands in 36/57 (63.2%) of cases, most commonly arising in the palate/maxillary gingiva (33.3%) and parotid gland (21.1%). Pathologic T category was evenly distributed (T1–2: 49.1%; T3–4: 50.9%), while nodal metastasis was uncommon (pN+: 17.5%). High-risk pathological features were frequent, including perineural invasion (PNI+) in 57.9% and close/positive margins in 38.6% (Table 1).

### 3.2. Treatment

All patients underwent primary surgical resection. Postoperative radiotherapy was administered in 41 patients (71.9%); no patient received concurrent chemotherapy. Among irradiated patients (*n* = 41), the median delivered dose was 70 Gy (IQR 60–70; range 50–74). The remaining 16 patients (28.1%) did not receive adjuvant radiotherapy. Adjuvant RT was preferentially delivered to patients with adverse pathological features, including PNI (68.3% with RT vs. 31.3% without RT; *p* = 0.017) and close/positive margins (51.2% with RT vs. 6.3% without RT; *p* = 0.002).

### 3.3. Follow-Up and Stat Us at Last Contact

Median follow-up for the entire cohort was 133 months (IQR 38–236; range 1–470). Among surviving patients, the median follow-up was 212 months (IQR 134–287), reflecting very prolonged observation. At last follow-up, 26 patients (45.6%) were alive without evidence of disease, one (1.8%) was alive with disease, 20 (35.1%) had died from HNAdCC, and 10 (17.5%) had died from other causes.

### 3.4. Survival Outcomes

**Figure 1:**
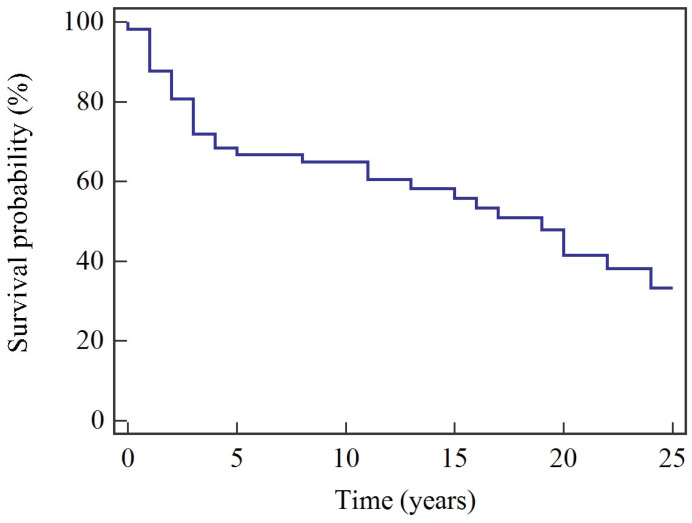
Kaplan–Meier overall survival (OS).

**Figure 2:**
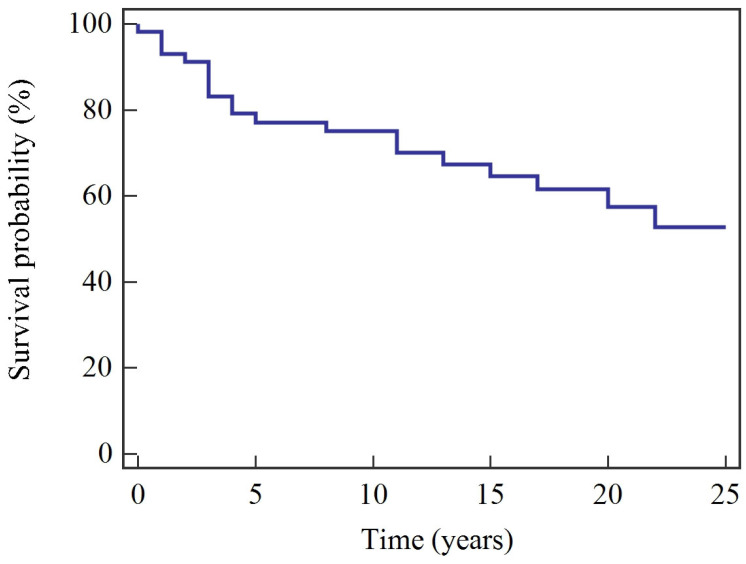
Kaplan–Meier cancer-specific survival (CSS).

Median overall survival (OS) was 224 months (18.7 years), while median cancer-specific survival (CSS) was not reached. Estimated 5-year OS and CSS were 68.4% and 78.9%, respectively; at 25 years, OS and CSS were 37.5% and 51.7% (Table 2). Kaplan–Meier survival curves are shown in Figure 1 and Figure 2.

### 3.5. Patterns and Timing of First Failure

**Figure 3:**
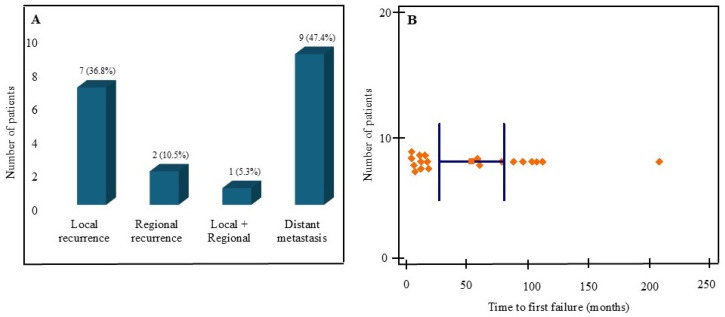
First failure patterns and timing. (**A**) First failure pattern among patients with documented first failure (*n* = 19). (**B**) Distribution of time to first failure (months).

Three patients (5.3%) had immediate local residual disease after primary treatment. In addition, 19 patients (33.3%) developed a documented first failure event. Among first failures (*n* = 19), distant metastasis was most common (*n* = 9, 47.4%), followed by local recurrence (*n* = 7, 36.8%), regional recurrence (*n* = 2, 10.5%), and combined local and regional recurrence (*n* = 1, 5.3%) (Figure 3A; Supplementary Table S1). The lung was the predominant first metastatic site (8/9, 88.9%). Median time to first failure was 14 months (IQR 8–88; range 0–204), but late failures were frequent: 7/19 (36.8%) occurred beyond 5 years and one beyond 10 years (Figure 3B). Salvage surgery was performed in 13 patients overall (22.8%) and in 11/19 (57.9%) patients with first failure. Across all salvage procedures (*n* = 13), 9 were performed for isolated local recurrence, 2 for isolated regional recurrence, 1 for combined locoregional recurrence, and 1 was a pulmonary metastasectomy for an isolated lung metastasis. At first failure, salvage was undertaken for all locoregional failures (7 local, 2 regional, 1 locoregional) and for 1/9 distant failures (isolated lung); the remaining first-failure distant metastases (8/9) were not resected because disease was multifocal/bilateral and/or accompanied by additional metastatic sites, limiting the expected benefit of metastasectomy.

### 3.6. Competing Risks and Causes of Death

**Figure 4:**
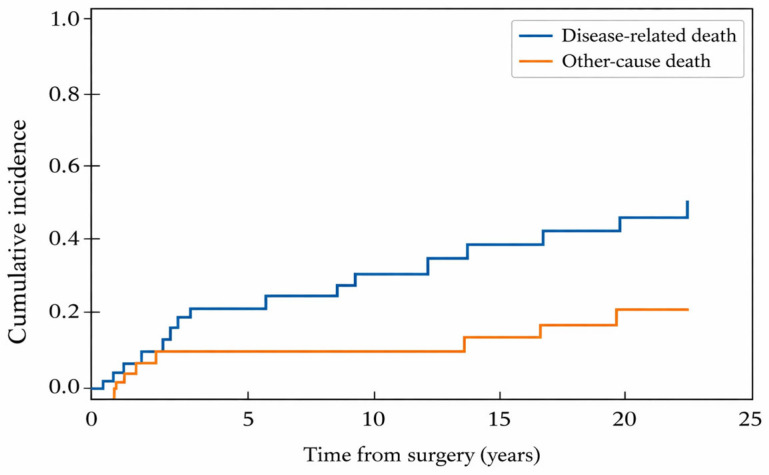
Competing-risks cumulative incidence of disease-related death and other-cause death.

In competing-risk analysis, the cumulative incidence of disease-related death increased steadily over time, reaching 41.7% at 25 years, whereas other-cause death remained 12.3% through 15 years and increased to 20.9% by 20 years (Table 2). The competing cumulative incidence functions for disease-related and other-cause death are shown in Figure 4.

### 3.7. Prognostic Factors

Univariable Cox regression results are provided in Supplementary Table S6. In multivariable analysis for OS, older age (per 10-year increase: HR 1.88, *p* < 0.001) and advanced T category (T3–4 vs. T1–2: HR 3.09, *p* = 0.006) remained independently associated with worse OS. In the multivariable CSS model, older age (per 10-year increase: HR 1.77, *p* = 0.010) and PNI (HR 5.50, *p* = 0.021) were independently associated with worse CSS, while T category was not statistically significant after adjustment (Table 3). Given the strong association between T category and PNI in our cohort, we performed a sensitivity analysis excluding PNI: in an age-adjusted CSS model, T3–4 disease remained significantly associated with worse CSS (HR 3.26, 95% CI 1.23–8.66; *p* = 0.018). Absence of adjuvant RT was not significantly associated with OS or CSS in univariable models (Supplementary Table S2).

### 3.8. Additional Analyses of Late-Event Dynamics

**Figure 5:**
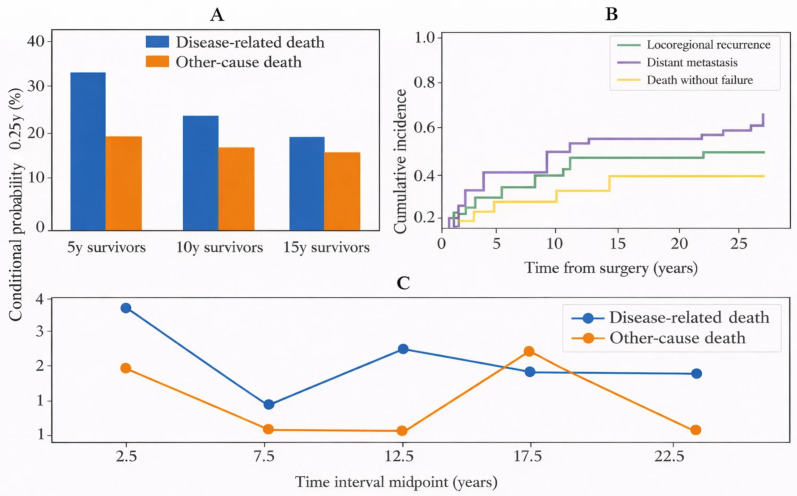
Additional analyses of late-event dynamics. (**A**) Conditional probability of death by cause to 25 years among 5-, 10-, and 15-year survivors. (**B**) Competing-risk cumulative incidence of first failure patterns and death without failure (*n* = 54; excluding immediate residual disease). (**C**) Piecewise incidence rates of disease-related and other-cause death per 100 person-years across 5-year intervals.

Conditional survival analyses (Supplementary Table S3; Figure 5A) showed that, among patients alive at 5 years, the conditional probability of surviving the next 5 years was high (conditional 5–10-year OS 94.8%). Nonetheless, late attrition persisted: among 5-year survivors, the conditional probability of disease-related death by 25 years remained 32.7%, compared with 12.5% for other-cause death; among 15-year survivors, conditional 25-year mortality was split between disease-related (17.4%) and other-cause (15.4%) death (Figure 5C). In competing-risk analysis of first failure patterns excluding immediate residual disease (*n* = 54), the 25-year cumulative incidence of locoregional recurrence and distant metastasis as first failure was 18.9% and 18.2%, respectively, while death without a documented first failure reached 24.9% (Supplementary Table S4; Figure 5B). In the 5-year landmark cohort (*n* = 35), the additional 5-year cumulative incidence of first failure was 18.2% (locoregional 12.0%, distant 6.2%). After first failure, median post-failure OS was 39 months, with substantially prolonged post-failure survival among patients undergoing salvage surgery (median 85 months) compared with those without salvage surgery (median 8 months) (Supplementary Table S5). Restricted mean survival time (RMST) to 25 years was 178.3 months (14.9 years), with large absolute differences by key risk features (Supplementary Table S6).

## 4. Discussion

In this single-institution cohort of surgically treated HNAdCC with follow-up spanning four decades, we observed the characteristic “long tail” of risk: 5-year outcomes were relatively favorable, yet both OS and CSS declined steadily over time, and clinically meaningful late failures accumulated well beyond conventional surveillance windows. In our cohort, OS decreased to approximately 37% at 25 years, while CSS remained near 52%, emphasizing that disease-related mortality continues to accrue for decades even among long-term survivors. Distant metastasis, predominantly pulmonary, was the most frequent first failure, and over one-third of first failures occurred beyond 5 years, highlighting that HNAdCC behaves less like a “cured/not cured” disease and more like a chronic oncologic condition requiring ongoing risk management.

Our survival estimates and failure patterns are consistent with contemporary pooled evidence, which also identifies distant metastasis as the dominant adverse outcome and demonstrates progressive attrition in survival beyond 10–15 years. A recent large meta-analysis (17,497 cases) reported 5-, 10-, 15-, and 20-year survival rates of roughly 74%, 49%, 42%, and 27%, respectively, and similarly emphasized distant metastasis as the most frequent unfavorable event [16]. Population-based analyses have also documented that long-term outcome estimates can differ substantially depending on whether overall or disease-specific endpoints are used, and that competing causes of death become increasingly influential with very long follow-up [20]. Against this background, our cohort adds value by providing detailed institutional data with unusually prolonged observation, explicit characterization of first-failure patterns, and additional time-dynamic risk estimates that are rarely reported in HNAdCC.

Locoregional control in our series was relatively favorable compared with distant control, with local recurrence occurring less often than distant metastasis as a first event and regional recurrence remaining uncommon. This likely reflects, at least in part, cohort selection and case mix: most tumors arose in major salivary glands or the palate/maxillary gingiva (sites generally more amenable to complete resection), sinonasal primaries comprised only 12.3% of cases, and patients treated with definitive primary radiotherapy for surgically unresectable disease were not included. This pattern supports the prevailing concept that local therapy can achieve meaningful control in many patients, while systemic dissemination drives late attrition. The role of postoperative radiotherapy (PORT) in HNAdCC remains primarily to improve local control rather than definitively improve long-term survival. A recent systematic review and meta-analysis comparing surgery alone with surgery plus PORT found a significant improvement in local control but no clear overall survival advantage at 5 or 10 years, noting the limitations of retrospective evidence and heterogeneity of included cohorts [21]. Current European guidance for salivary gland cancers similarly recommends adjuvant radiotherapy in the presence of high-risk features (e.g., advanced T category, close or positive margins, and perineural invasion), reflecting a consensus that improved locoregional control is clinically meaningful even when a survival benefit is difficult to demonstrate in non-randomized data [22]. In our cohort, treatment selection and the long accrual period preclude causal inference regarding PORT; however, the observed distribution of failures aligns with the concept that durable local control must be paired with strategies addressing long-term metastatic risk.

Among baseline clinicopathologic factors, perineural invasion (PNI) emerged as a particularly strong predictor of disease-related mortality in multivariable analysis, while older age and advanced T category were associated with worse overall survival. Advanced stage has consistently been associated with worse survival in ACC across historical, contemporary, and site-specific cohorts (e.g., Spiro et al.; Mendenhall et al.; Marcinow et al.; Mays et al.), and population-based analyses similarly identify stage as a major determinant of outcome [7,8,23,24,25]. In our cohort, the prognostic impact of T category on CSS was attenuated after adjustment for PNI, likely reflecting substantial overlap between these variables and limited power; nevertheless, sensitivity analyses supported a persistent association between T3–4 disease and worse CSS when PNI was not included. These findings are biologically plausible and clinically actionable. PNI is a hallmark of HNAdCC and likely reflects both local infiltrative potential and a broader aggressive phenotype, which may translate into an increased propensity for late relapse. Notably, nodal events were infrequent in our cohort, paralleling contemporary evidence that clinically node-negative HNAdCC has a relatively low rate of occult nodal metastasis and that elective neck treatment should be individualized rather than routinely applied [26,27]. Together, these factors support a risk-adapted approach to counseling and surveillance, with particular intensification for patients with PNI and/or advanced T stage.

A major innovation of the present study is the explicit quantification of late-event dynamics using competing risks and conditional risk estimation. In conventional Kaplan–Meier analyses, late attrition can be misinterpreted if competing deaths are not considered; therefore, we estimated cumulative incidence functions for disease-related and other-cause death. By 25 years, disease-related death constituted the larger competing component, but other-cause mortality was non-negligible, reflecting the inevitability of aging-related competing risks during multi-decade observation. More clinically important, our conditional analyses demonstrate that surviving a given number of years does not eliminate risk in HNAdCC: among 5-year survivors, the conditional probability of disease-related death by 25 years remained approximately one-third, and even among 15-year survivors, conditional long-term mortality remained meaningfully split between disease-related and other-cause death. This time-dynamic framing is highly relevant for survivorship counseling, as it communicates residual risk in a way that is intuitive for patients and clinicians and directly informs follow-up planning beyond arbitrary 5-year milestones.

Complementing hazard-based modeling, we also used restricted mean survival time (RMST) to express long-term outcome differences on an absolute time scale. RMST is increasingly recommended as a robust and clinically interpretable alternative when proportional hazards are questionable or when late events dilute the interpretability of hazard ratios [28]. In our cohort, RMST up to 25 years showed large absolute survival-time differences associated with key risk factors: patients without PNI and those with T1–2 disease experienced an advantage of 7–8 years of life lived over the 25-year horizon compared with their higher-risk counterparts. These findings reinforce that, despite the indolent reputation of HNAdCC, adverse pathologic features translate into substantial long-term life-years lost—an argument for treatment intensification, careful counseling, and structured long-term follow-up in high-risk patients.

Practical surveillance proposal based on our conditional risks: Our conditional competing-risk estimates indicate that clinically meaningful disease-related mortality persists even after 5 and 10 years, arguing against de-escalation to symptom-driven follow-up in HNAdCC survivors. We propose a pragmatic, risk-adapted schedule aligned with European guidance: clinical assessment every 3–4 months during years 1–2, every 6 months during years 3–5, and annually thereafter, with continued long-term follow-up beyond 10 years [22]. Given the predominance of pulmonary metastasis and the sustained conditional risk observed in our cohort, annual chest imaging (preferably CT) is reasonable for at least 10 years and can be continued beyond that in patients with high-risk features (PNI, T3–4, close or positive margins) or by patient preference [18,22]. For the primary site, periodic MRI can be considered in the earlier years and selectively thereafter based on risk and symptoms, particularly in tumors with perineural spread or skull-base proximity [22]. Finally, surveillance intensity should be individualized according to comorbidity and competing mortality risk, recognizing that other-cause death becomes progressively more relevant in later decades.

Post-failure outcomes in our cohort also have actionable implications. Median survival after first failure remained measured in years, and a subset of patients, particularly those with locoregional failure amenable to salvage, achieved prolonged post-failure survival. Importantly, the marked post-failure survival difference by salvage status should not be interpreted causally: patients undergoing salvage predominantly had limited, resectable locoregional recurrence (and one oligometastatic lung case), whereas most non-salvage patients had disseminated distant metastases not amenable to curative-intent resection; thus salvage surgery likely functions as a marker of low disease burden and favorable biology at failure. While selection biases are unavoidable, these data support an “active management” approach when failures are detected at an oligometastatic or surgically salvageable stage. This rationale is increasingly relevant because systemic options for recurrent or metastatic HNAdCC remain limited but are evolving. Phase II evidence supports VEGFR-directed strategies such as lenvatinib and combinations such as axitinib plus avelumab, which can provide disease control in progressive disease but require careful toxicity management [29,30]. A recent systematic review and meta-analysis of VEGFR inhibitors reported measurable objective responses and clinically relevant disease stabilization, underscoring the role of antiangiogenic approaches as a backbone for systemic control in selected patients [31]. At the same time, molecular profiling is becoming more informative; recent genomic meta-analytic work highlights recurrent alterations (e.g., MYB-pathway changes and NOTCH-related alterations) and supports broader genomic testing in advanced disease to identify candidates for targeted or trial-based therapies [32].

Advances in radiation technology may further improve therapeutic ratios. Recent proton therapy series in HNAdCC demonstrate high locoregional control with acceptable toxicity profiles, supporting its consideration when dose escalation and organ-at-risk constraints are critical, particularly for skull base or perineural extension scenarios [33]. Although our cohort spans eras with heterogeneous imaging and radiotherapy techniques, these contemporary data provide a plausible pathway to improving local control while maintaining function, which remains a key goal given the long survivorship horizon of many HNAdCC patients.

This study has limitations inherent to its retrospective design, small sample size, and long accrual period: pathology reporting standards, imaging availability, and adjuvant treatment strategies changed over decades; cause-of-death assignment may be imperfect; and detailed systemic therapy data were limited. In addition, post-failure comparisons by salvage surgery are inherently subject to selection and time-dependent biases, because salvage candidacy reflects resectability, disease burden, and patient fitness at recurrence. We acknowledge that T-category assignment for tumors of the hard palate/maxillary gingiva may differ depending on whether oral cavity versus maxillary sinus staging is applied; in this study we used oral cavity staging based on primary epicenter, which may have contributed to the observed T-category distribution in this subgroup. Nonetheless, the principal strengths are the exceptionally long follow-up, simultaneous reporting of OS and CSS, explicit first-failure pattern characterization, and the use of competing-risk, conditional, and RMST approaches to quantify clinically meaningful late-event dynamics. Together, these results reinforce that HNAdCC requires lifelong, risk-adapted survivorship strategies and that time-dynamic risk metrics can make long-term counseling and follow-up planning more evidence-based and clinically intuitive.

## 5. Conclusions

Head and neck adenoid cystic carcinoma poses a persistent long-term risk, with clinically significant late recurrences and increasing competing mortality extending well beyond standard follow-up periods. In this 40-year surgical cohort, distant, primarily pulmonary metastasis was the main long-term threat, and baseline factors such as advanced T category and perineural invasion identified patients at highest risk. Conditional and RMST-based analyses indicate that considerable disease-related risk remains even among long-term survivors, supporting lifelong, risk-adapted surveillance with particular focus on detecting distant metastases.

## Figures and Tables

**Table 1:** Baseline clinicopathologic characteristics and treatment. Palate/maxillary gingiva primaries were staged according to AJCC 8 oral cavity criteria.

Variable	Value
Demographics	
Patients, n	57
Age, years	53.2 ± 13.9; median 54 (43–63)
Female sex, n (%)	30 (52.6%)
Tumor characteristics	
Origin, n (%)	
Major salivary glands	21 (36.8%)
Minor salivary glands	36 (63.2%)
Primary site, n (%)	
Palate/Maxillary gingiva	19 (33.3%)
Parotid	12 (21.1%)
Oral cavity	10 (17.5%)
Submandibular	9 (15.8%)
Sinonasal (maxillary/ethmoid/nasal)	7 (12.3%)
T category (grouped), n (%)	
T1–2	28 (49.1%)
T3–4	29 (50.9%)
Pathologic N category, n (%)	
pN0	47 (82.5%)
pN1	6 (10.5%)
pN2	3 (5.3%)
pN3	1 (1.8%)
Pathological stage, n (%)	
I	18 (31.6%)
II	8 (14.0%)
III	11 (19.3%)
IVa	14 (24.6%)
IVb	6 (10.5%)
Largest tumor dimension, mm	28.4 ± 15.8; median 25 (18–40)
Perineural invasion (PNI+), n (%)	33 (57.9%)
Perivascular invasion (PVI+), n (%)	18 (31.6%)
Positive/Close surgical margins, n (%)	22 (38.6%)
Extranodal extension (ENE+), n (%)	5 (8.8%)
Treatment	
Adjuvant RT, n (%)	41 (71.9%)
No adjuvant RT, n (%)	16 (28.1%)
RT dose, Gy (irradiated patients, *n* = 41)	70 (60–70); range 50–74

**Table 2:** Long-term survival and competing-risk estimates.

Variable	Value
Kaplan–Meier estimates	
OS at 5 years	68.4% (95% CI 54.7–78.8)
OS at 10 years	64.9% (95% CI 51.0–75.7)
OS at 15 years	55.8% (95% CI 41.3–68.0)
OS at 20 years	41.2% (95% CI 26.4–55.5)
OS at 25 years	37.5% (95% CI 22.6–52.3)
CSS at 5 years	78.9% (95% CI 65.1–88.7)
CSS at 10 years	74.8% (95% CI 60.5–84.6)
CSS at 15 years	64.3% (95% CI 48.5–76.4)
CSS at 20 years	56.9% (95% CI 39.8–70.8)
CSS at 25 years	51.7% (95% CI 33.4–67.2)
Median OS, months	224 (18.7 years)
Median CSS	Not reached
Competing risks (Aalen–Johansen)	
CIF disease-related death at 5 years	19.3%
CIF other-cause death at 5 years	12.3%
CIF disease-related death at 10 years	22.9%
CIF other-cause death at 10 years	12.3%
CIF disease-related death at 15 years	31.9%
CIF other-cause death at 15 years	12.3%
CIF disease-related death at 20 years	37.9%
CIF other-cause death at 20 years	20.9%
CIF disease-related death at 25 years	41.7%
CIF other-cause death at 25 years	20.9%

**Table 3:** Multivariable Cox regression analysis for overall survival (OS) and cancer-specific survival (CSS).

Variable	OS HR (95% CI)	*p*	CSS HR (95% CI)	*p*
Age (per 10 years)	1.88 (1.36–2.59)	<0.0001	1.77 (1.15–2.72)	0.010
T3–4 vs. T1–2	3.09 (1.38–6.90)	0.006	1.37 (0.45–4.19)	0.581
Perineural invasion (PNI+)	—	—	5.50 (1.30–23.31)	0.021

Multivariable models included the covariates shown. The OS model included age and T category; the CSS model included age, T category, and PNI.

## Data Availability

All data generated or analyzed during this study are included in this published article.

## Supplementary Materials

The following supporting information can be downloaded at: https://www.mdpi.com/article/10.3390/cancers18050833/s1, Figure S1: STROBE-style flow diagram of cohort assembly and analytic datasets; Table S1: Follow-up, status at last contact, and patterns/timing of first failure; Table S2: Univariable Cox regression results for overall survival (OS) and cancer-specific survival (CSS); Table S3: Conditional survival and conditional long-term mortality; Table S4: Competing-risk analysis of first failure patterns (excluding immediate residual disease) and 5-year landmark analysis; Table S5: Post-failure survival (time from first failure to death or last follow-up); Table S6: Restricted mean survival time (RMST) for overall survival up to 25 years.

## Abbreviations

The following abbreviations are used in this manuscript:

AdCC	Adenoid cystic carcinoma
AJCC	American Joint Committee on Cancer
HNAdCC	Head and neck adenoid cystic carcinoma
CSS	Cancer-specific survival
CIF	Cumulative incidence function
PNI	Perineural invasion
PVI	Perivascular invasion
ENE	Extranodal extension
RT	Radiotherapy
PORT	Postoperative radiotherapy
CT	Computed tomography
MRI	Magnetic resonance imaging
Gy	Gray
