# Supplementary material for: Persistent Long-Term Risk After Primary Surgery for Head and Neck Adenoid Cystic Carcinoma: Competing-Risk and Conditional Estimates
Source: Cancers (Basel). 2026 Mar 4;18(5):833. doi: 10.3390/cancers18050833 (PMC12984544; doi:10.3390/cancers18050833)
Supplement: Supplementary file 1 [file cancers-18-00833-s001.zip › cancers-4171558-supplementary.pdf]

Supplementary Material

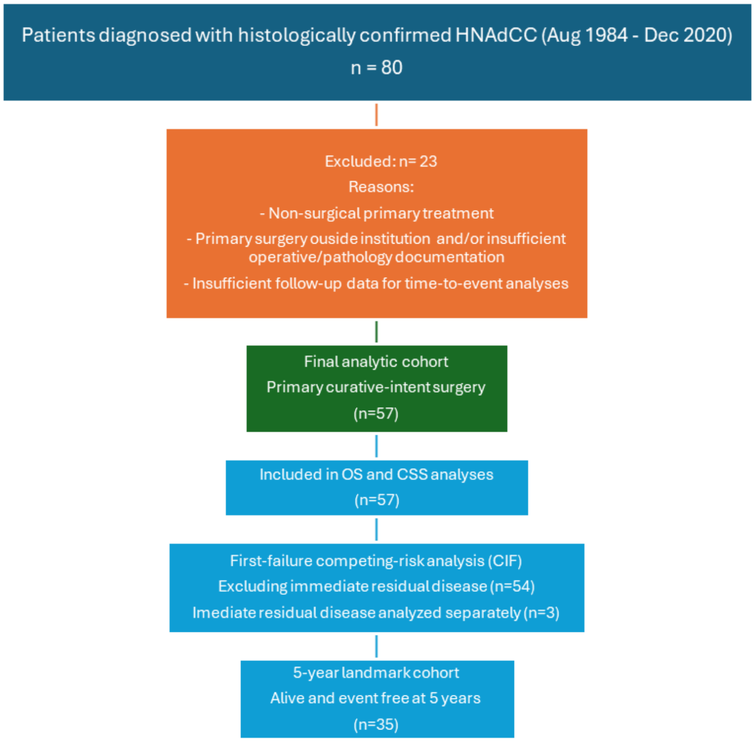

Figure S1. STROBE-style flow diagram of cohort assembly and analytic datasets.

Table S1. Follow-up, status at last contact, and patterns/timing of first failure.

Variable	Value
**Follow-up and status**	
**Follow-up, months**	147.4 ± 111.3; median 133 (38–236); range 1–470
**Follow-up among survivors, months**	217.2 ± 94.2; median 212 (134–287)
**Status at last follow-up: Alive, no evidence of disease**	26 (45.6%)
**Status at last follow-up: Alive with disease**	1 (1.8%)
**Status at last follow-up: Dead of disease**	20 (35.1%)
**Status at last follow-up: Dead of other causes**	10 (17.5%)
**Deaths (all-cause), n (%)**	30 (52.6%)
**Cancer-specific deaths, n (%)**	20 (35.1%)
**Deaths from other causes, n (%)**	10 (17.5%)
**Recurrence/metastasis**	Recurrence/metastasis
**Any first failure (local/regional/distant), n (%)**	19 (33.3%)
**Local residual disease after primary treatment, n (%)**	3 (5.3%)
**Local recurrence (any), n (%)**	8 (14.0%)
**Regional recurrence (any), n (%)**	3 (5.3%)
**Distant metastasis as first failure, n (%)**	9 (15.8%)
**First failure pattern among failures (n=19)**	
**Local recurrence**	7 (36.8%)
**Regional recurrence**	2 (10.5%)
**Local + regional**	1 (5.3%)
**Distant metastasis**	9 (47.4%)
**First distant metastasis site (n=9)**	
**Lung**	8 (88.9%)
**Bone**	1 (11.1%)
**Time to first failure, months (n=19)**	49.5 ± 55.2; median 14 (8–88); range 0–204
**Late failures >5 years, n (%)**	7 (36.8%)
**Late failures >10 years, n (%)**	1 (5.3%)
**Second local recurrence, n (%)**	4 (7.0%)
**Second distant metastasis, n (%)**	1 (1.8%)
**Residual local disease after treatment of first recurrence, n (%)**	1 (1.8%)
**Any salvage surgery (recurrence/residual/metastasis), n (%)**	13 (22.8%)
**Salvage surgery among failures (n=19), n (%)**	11 (57.9%)

Table S2. Univariable Cox regression results for overall survival (OS) and cancer-specific survival (CSS).

Variable	OS HR (95% CI)	p	CSS HR (95% CI)	*p*
**Age (per 10 years)**	1.89 (1.38–2.60)	<0.001	1.68 (1.14–2.48)	0.008
**Female vs male**	1.15 (0.55–2.39)	0.705	1.36 (0.55–3.37)	0.502
**Minor vs major salivary gland origin**	1.88 (0.84–4.25)	0.127	2.76 (0.92–8.30)	0.070
**T3–4 vs T1–2**	3.28 (1.49–7.20)	0.003	3.37 (1.28–8.83)	0.014
**pN+ vs pN0**	2.38 (1.01–5.60)	0.048	2.04 (0.67–6.16)	0.209
**Perineural invasion (PNI+)**	2.85 (1.26–6.45)	0.012	5.95 (1.73–20.44)	0.005
**Perivascular invasion (PVI+)**	2.08 (1.00–4.34)	0.051	2.66 (1.09–6.45)	0.031
**Positive margins**	1.06 (0.51–2.24)	0.873	1.22 (0.50–2.99)	0.661
**Extranodal extension (ENE+)**	5.16 (1.69–15.76)	0.004	4.14 (0.89–19.28)	0.070
**Mandible resection**	2.20 (0.94–5.15)	0.070	1.95 (0.64–5.87)	0.238
**No adjuvant RT vs adjuvant RT**	1.81 (0.87–3.77)	0.112	1.52 (0.60–3.82)	0.374
**Tumor size (per 10 mm)**	1.46 (1.12–1.91)	0.005	1.50 (1.10–2.06)	0.011

Table S3. Conditional survival and conditional long-term mortality.

Interval (years)	Conditional OS (%)	Conditional CSS (%)
**0–5**	68.4	78.9
**5–10**	94.8	94.8
**10–15**	86.0	86.0
**15–20**	73.9	88.4
**20–25**	90.9	90.9

Landmark survivors	Conditional disease-related death by 25y (%)	Conditional other-cause death by 25y (%)
**Alive at 5 years**	32.7	12.5
**Alive at 10 years**	29.0	13.2
**Alive at 15 years**	17.4	15.4

Table S4. Competing-risk analysis of first failure patterns (excluding immediate residual disease) and 5-year landmark analysis.

Time (years)	Locoregional recurrence CIF (%)	Distant metastasis CIF (%)	Death without failure CIF (%)	Event-free (%)
**5**	11.1	11.1	13.0	64.8
**10**	18.9	15.1	14.9	51.1
**15**	18.9	15.1	14.9	51.1
**20**	18.9	18.2	24.9	38.1
**25**	18.9	18.2	24.9	38.1

5-year landmark cohort (n = 35)	Locoregional CIF (%)	Distant CIF (%)	Death without failure CIF (%)	Event-free (%)
**10 years (additional 5 years)**	12.0	6.2	2.9	78.8
**20 years (additional 15 years)**	12.0	10.9	18.4	58.7

Table S5. Post-failure survival (time from first failure to death or last follow-up).

Group	n	Median post-failure OS (months)	Post-failure OS at 5 years (%)	Post-failure OS at 10 years (%)
**All first failures**	19	39	41.4	29.6
**Distant metastasis**	9	39	29.6	14.8
**Locoregional failure**	10	37	50.0	40.0
**Salvage surgery**	11	85	54.5	43.6
**No salvage surgery**	8	8	25.0	12.5

Table S6. Restricted mean survival time (RMST) for overall survival up to 25 years.

Comparison	Group A	Group B	RMST A (months)	RMST B (months)	Difference (months)	95% CI (months)
**PNI**	PNI–	PNI+	228.8	140.5	88.3	25.3 to 146.9
**T category**	T1–2	T3–4	226.1	131.3	94.9	36.0 to 151.8
